# Optic Disc Swelling in Cancer Patients: Etiology and Implications

**DOI:** 10.3390/jcm12227140

**Published:** 2023-11-17

**Authors:** Yacoub A. Yousef, Isra M. Sid Ahmed, Danah Kanj Ahmad, Mona Mohammad, Hala Makahleh, Reem AlJabari, Fawzieh Alkhatib, Mario Damiano Toro, Robert Rejdak, Mustafa Mehyar, Ibrahim Alnawaiseh

**Affiliations:** 1 Department of Surgery/Ophthalmology, King Hussein Cancer Centre (KHCC), Amman 11941, Jordan; isra.sidahmed@gmail.com (I.M.S.A.); ra.11229@khcc.jo (R.A.); mustafamehyar@hotmail.com (M.M.); i-nawaiseh@hotmail.com (I.A.); 2 Alzarga Eye Center, Khartoum 11115, Sudan; 3 Chair and Department of General and Pediatric Ophthalmology, Medical University of Lublin, 20-079 Lublin, Poland; 4 Eye Clinic, Public Health Department, University of Naples Federico II, 80131 Naples, Italy

**Keywords:** optic disc swelling, papilledema, chemotherapy, infiltration, Jordan

## Abstract

Purpose: To analyze the etiology and implications of optic disc swelling in cancer patients treated at a specialized tertiary cancer center in Jordan. Methods: This was a retrospective study of all cancer patients who had optic disc swelling between January 2019 and December 2020 at King Hussein Cancer Center (KHCC). Patients’ data included age, sex, laterality, visual acuity, and the underlying cause and management for the optic disc swelling. Results: Optic disc swelling was present in 58 cancer patients (96 eyes), with 38 (65%) having bilateral involvement. Among these, 33 (57%) were female, and 43 (74%) were ≤40 years old. At diagnosis, 58 (63%) eyes had a best-corrected visual acuity (BCVA) better than 0.5, improving to 73 (76%) eyes at the last follow-up. High intracranial pressure (ICP) was the most common primary cause (30 patients/52%), followed by tumor infiltration of the optic nerve (10 patients/17%), optic nerve compression (7 patients/12%), and optic nerve inflammation (5 patients/9%). Four patients had pseudopapilledema. Among the 30 patients with high ICP, CNS tumors were predominant (21 patients/70%), with only 3 having idiopathic intracranial hypertension. Medications, including ATRA (All-Trans Retinoic Acid) and systemic steroids, contributed to increased ICP in six patients (20%). BCVA was less than 0.5 in all eyes (100%) affected by tumor infiltration, optic nerve inflammation, and ischemic optic neuropathy, while only eight eyes (14%) with optic disc swelling due to elevated ICP had a BCVA less than 0.5 (*p* < 0.0001). Management included steroids (53 patients/91%), acetazolamide (30 patients/52%), chemotherapy (20 patients/34%), radiation therapy (13 patients/22%), frequent lumbar punctures (12 patients/21%), and surgery (28 patients/48%). Visual acuity improved in 40 eyes (42%), with only 4 eyes (4%) experiencing deterioration. At a 12-month median follow-up period, 11 (19%) patients were dead, 10 (10%) eyes had poor vision (BCVA less than 0.1), and 21 (22%) eyes had BCVA 0.5 or better. Conclusions: Various underlying pathologies can induce optic disc swelling in cancer patients, a grave condition capable of causing vision loss. Notably, tumor infiltration of the optic nerve tends to result in more profound visual impairment compared to papilledema due to elevated ICP. Timely detection is crucial, and immediate symptomatic treatment followed by addressing the underlying cause is essential to prevent irreversible optic nerve damage and vision loss in cancer patients.

## 1. Introduction

Optic nerve swelling, also referred to as optic disc swelling, is a medical condition characterized by the enlargement and inflammation of the optic disc, and this could be unilateral or bilateral. Optic nerve swelling is distinguished by features such as engorgement of the optic disc, the presence of edema, and changes in the disc’s margins. When bilateral optic disc swelling occurs in conjunction with increased intracranial pressure (ICP), it is specifically termed “papilledema” [1]. Papilledema stands out as the most frequent cause of optic disc swelling in patients who are under 50 years old, including the pediatric population. In the United States, idiopathic intracranial hypertension (IIH) ranks as the primary cause of papilledema. Other factors contributing to bilateral optic disc swelling encompass conditions like infiltrative optic neuropathy, toxic optic neuropathy, and malignant hypertension [1,2,3]. When optic disc swelling affects only one eye, it may be attributed to various underlying causes, such as optic neuritis (ON), non-arteritic anterior ischemic optic neuropathy (NA-AION), compressive optic neuropathy, retinal vein occlusion, and diabetic papillopathy. Among Caucasians, anterior ischemic optic neuropathy has been documented as the most prevalent cause of unilateral optic disc swelling [4,5,6,7].

Cancer represents a global health challenge with significant implications, affecting millions of individuals and resulting in considerable morbidity and mortality. While the primary focus of cancer care revolves around the management of primary tumors, it is crucial not to underestimate the importance of addressing ocular complications in cancer patients. One notable ocular concern in this context is the occurrence of optic nerve swelling, which carries the potential for visual impairment and can substantially impact the overall quality of life for those individuals affected.

In cancer patients, apart from the previously mentioned factors, optic disc swelling can be attributed to several specific causes. These include increased intracranial pressure (ICP), which can arise from both primary and metastatic tumors affecting the brain. Additionally, certain medications used in cancer treatment can induce this condition, and in some cases, the tumor itself may directly infiltrate the optic nerve, as observed in patients with leukemia and lymphoma [8,9,10,11].

Understanding the occurrence of optic nerve swelling in cancer patients holds significant importance for multiple reasons. It provides insights into the ocular issues experienced by individuals dealing with cancer. Furthermore, the presence of optic nerve swelling can serve as an indicator of increased intracranial pressure (ICP), necessitating further examination to uncover potential underlying brain-related issues, and there is a risk of irreversible vision loss if immediate intervention is not pursued. The primary objective of this study is to investigate the causes and clinical presentations of optic disc swelling in cancer patients receiving care at a specialized tertiary cancer center.

## 2. Methods

Approval for this study was obtained from the Institutional Review Board at King Hussein Cancer Center (20KHCC58). This research comprised a retrospective clinical case series involving 58 cancer patients who exhibited optic disc swelling. Data for this study were derived from the medical records of cancer patients who had received treatment at King Hussein Cancer Center (KHCC). Inclusion criteria encompassed patients who had been diagnosed with optic disc swelling during their visits to the ophthalmology clinic between January 2019 and December 2020.

To obtain the necessary information, a thorough examination of the medical records of included patients was conducted. Patient-specific data, including age, gender, affected eye(s), initial visual acuity at the time of diagnosis, visual acuity documented during their last visit, color vision assessment at diagnosis, and color vision assessment at their final visit, were collected. In addition, color fundus photos, clinical records such as brain computed tomography (CT) and brain magnetic resonance imaging (MRI) reports, as well as laboratory reports from lumbar punctures (LPs) were reviewed to confirm the diagnosis of optic disc swelling and to ascertain the underlying cause of this condition. The initial medical interventions administered to patients upon their diagnosis of optic nerve swelling were also documented. Patients were actively monitored for a minimum duration of six months following the onset of optic disc swelling, or until any unfortunate demise occurred during the follow-up period. The clinical diagnosis of optic disc swelling was established through indirect ophthalmoscopy when there was an elevation in the disc surface or blurred disc margins, and fundus photos were documented when the patient was cooperative with the photographer.

Inclusion criteria included all cancer patients who presented with optic disc edema during the study period, which was documented by the attending ophthalmologist. Patients with congenital optic disc anomalies and those with dense media where the optic nerve head could not be assessed with accuracy were excluded from the study. Descriptive analysis of the collected data was conducted employing basic statistical measures such as mean, median, and range.

## 3. Results

During the study period, optic disc swelling was detected in 58 cancer patients, affecting a total of 96 eyes. Among these patients, 34% (20 patients) had primary brain tumors, 19% (11 patients) had breast cancer (including 3 with brain metastasis), 14% (8 patients) had leukemia, 9% (5 patients) had lymphoma, 5% (3 patients) had nasopharyngeal tumors, 5% (3 patients) had lung cancer (including 1 with brain metastasis), 5% (3 patients) had colorectal cancer (including 1 with brain metastasis), 2% (1 patient) had a renal tumor, 3% (2 patients) had retinoblastoma, 2% (1 patient) had an ovarian tumor, and 2% (1 patient) had choroidal melanoma.

Among these patients, 38 (66%) had bilateral optic nerve involvement, while 8 patients had right-sided and 12 had left-sided involvement. Two patients in this series were single-eyed as the other eye was enucleated for retinoblastoma. Thirty-three (57%) were female, while the male population accounted for twenty-five patients (43%). The age range in the study spanned from 1 to 80 years, with an average age of 38 years. The age distribution is provided in Table 1; nevertheless, it is noteworthy that 43 patients (74%) were aged 40 years or younger.

We were able to assess visual acuity at the initial presentation for 56 patients (92 eyes). Among these, 58 eyes (63%) exhibited a best-corrected visual acuity (BCVA) of 0.5 or better at the time of diagnosis; conversely, visual acuity was recorded as counting fingers or worse for 17 eyes (18%) (Table 1). At the last follow-up, visual acuity was assessed for all 96 eyes. Among these, 73 eyes (76%) demonstrated a visual acuity of 0.5 or better, while 10 eyes (10%) had a visual acuity of counting fingers or less (Table 1). Color vision assessments were conducted for 78 eyes at the time of diagnosis and 96 eyes at the last follow-up. The study’s findings revealed that normal color vision was observed in 43 eyes (55%) at the time of diagnosis. Furthermore, at the follow-up examination, 61 eyes (64%) exhibited normal color vision (Table 1).

**Figure 1:**
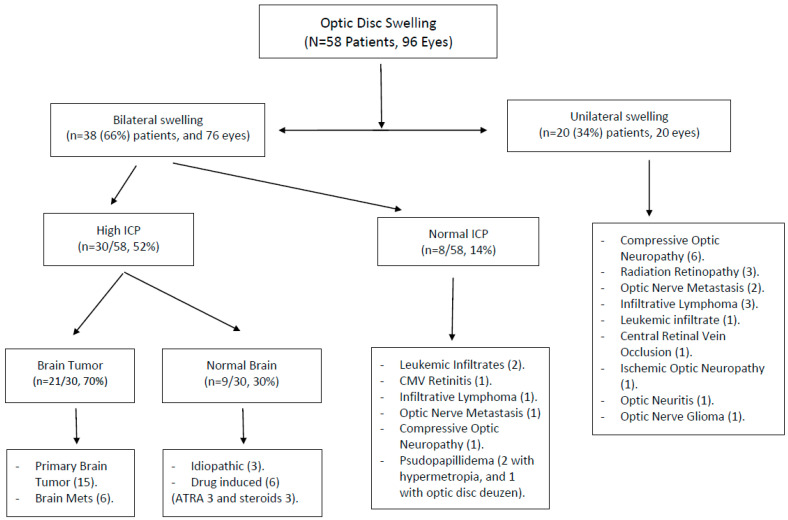
PRISMA flow diagram showing the laterality of optic disc swelling (unilateral vs. bilateral) and the distribution of underlying causes for each subgroup.

**Figure 2:**
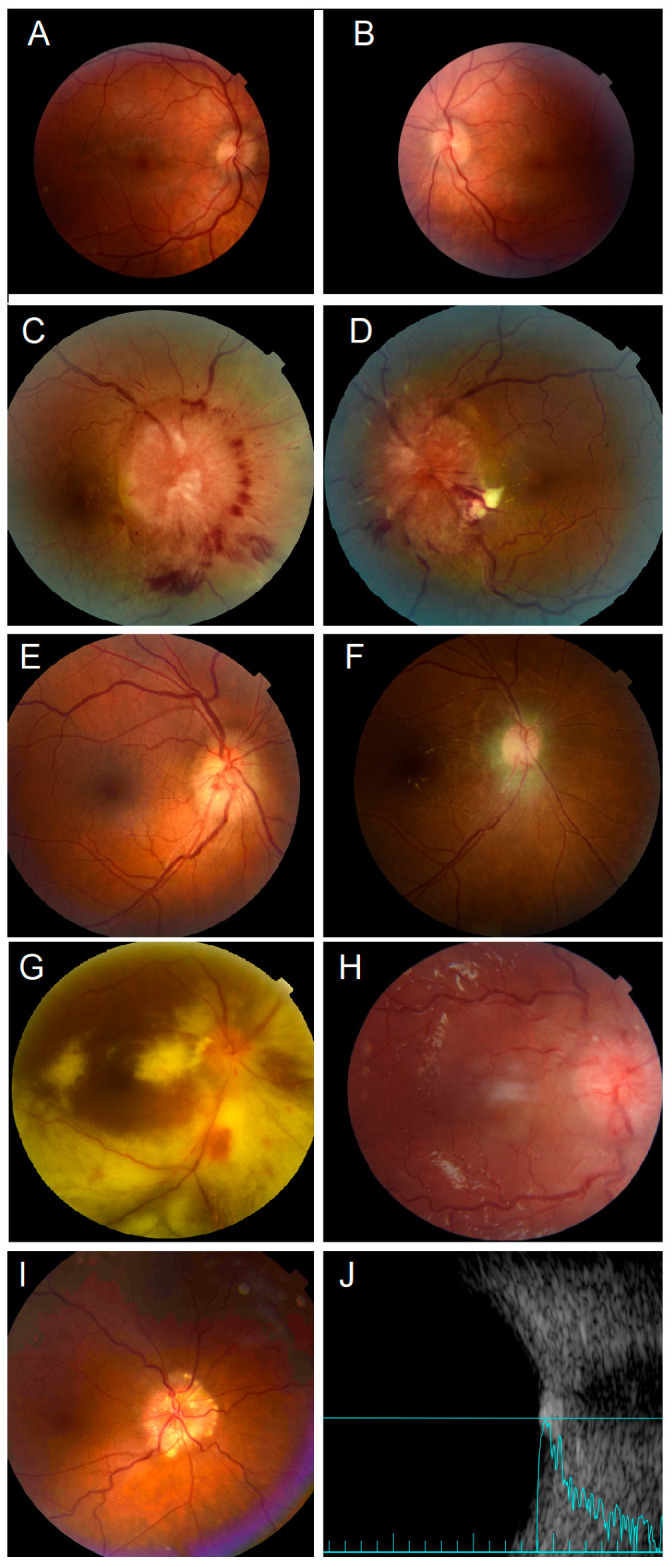
Fundus photos for optic disc swelling for different underlying diseases. (**A**,**B**) Bilateral optic disc swelling secondary to increased intracranial pressure. (**C**,**D**) Bilateral optic nerve metastasis in patients with metastatic breast cancer. Leukemia patient with optic nerve head leukemic infiltrate before (**E**) and after (**F**) treatment. (**G**) Patient with CMV retinitis. (**H**) Optic nerve metastasis in a child with contralateral retinoblastoma. (**I**) A patient with optic disc swelling and normal optic nerve functions was found to have optic disc drusen confirmed by hyperreflectivity in B-scan (**J**).

The most common cause of optic disc swelling was elevated intracranial pressure (ICP), accounting for 30 patients (52%). This was followed by tumor infiltration of the optic nerve, observed in 10 patients (17%), optic nerve compression, which was documented in 7 patients (12%), and optic nerve inflammation, noted in 5 patients (9%). The inflammatory causes encompassed conditions such as radiation retinopathy (in three patients), idiopathic optic neuritis (in one patient), and cytomegalovirus (CMV) retinitis (in one patient). Additionally, two patients presented with ischemic optic neuropathy; one patient exhibited central retinal vein occlusion (CRVO), and another had anterior ischemic optic neuropathy (AION). Four (7%) patients had pseudopapilledema; three had hyperopia; and one patient’s optic nerve swelling was attributed to the presence of optic nerve drusen (Table 2). The distribution of causes for optic disc swelling based on laterality is summarized in Figure 1. Figure 2 shows fundus photos for examples of different causes of optic disc swelling in this series.

Among the 30 patients diagnosed with elevated intracranial pressure (ICP), a detailed classification was conducted based on the underlying causes. Among these 30 cases, 14 individuals (47%) were females, while 16 (53%) were males. Thirteen patients (43%) of this subgroup were 20 years old or younger, and notably, all of them (100%) exhibited bilateral optic disc swelling. Five patients (17%) displayed normal optic nerve functions, including 20/20 vision (1.0) and normal color vision, seventeen (57%) had a visual acuity between 0.5 and 1.0, and eight (14%) patients had a visual acuity less than 0.5.

The predominant etiological factor in this group was central nervous system (CNS) tumors, affecting 21 patients (70%). Within this subgroup, 16 patients (76%) had primary CNS tumors, while 5 patients (24%) presented with metastatic CNS tumors. Additionally, medications were identified as contributing to increased ICP in six patients (20%). Among these medication-induced cases, three were attributed to ATRA (All-Trans Retinoic Acid), and the remaining three were due to systemic steroid use. The remaining three patients had idiopathic causes for their elevated ICP (Table 2). A lumbar puncture was carried out for all patients with suspected high ICP. The pathology of the CSF confirmed the diagnosis in three patients with leukemia infiltration of the optic nerve, in four patients with CNS lymphoma infiltrating the optic nerve, and in three patients with metastasis to the optic nerve.

At diagnosis, the best corrected visual acuity was worse than 0.5 in eight (14%) of the eyes with optic disc swelling secondary to high ICP, while all (100%) eyes with tumor infiltration of the optic nerve, optic nerve inflammation, and ischemic optic neuropathy had a visual acuity less than 0.5 (*p* < 0.0001) at presentation (Table 2).

Overall, in terms of management modalities for optic nerve swelling, the study reported that 53 patients (91%) (all patients except for the four patients who had pseudopapilledema and the one with CMV retinitis) initiated treatment with systemic steroids and/or Acetazolamide, which continued until the underlying cause was addressed. Steroids were used in 53 patients (91%), Acetazolamide in 30 patients (52%), chemotherapy treatment in 20 (34%) patients (including 2 intrathecal and 20 systemic), radiation therapy in 13 (22%) patients, frequent lumbar punctures in 12 (21%) patients, and surgery (tumor excision ± Ventriculoperitoneal (VP) shunt) in 28 (48%) patients. Thereafter, an improvement in visual acuity was observed in 40 eyes (42%), while vision remained stable in 52 (54%) eyes. Only four eyes (4%) experienced a deterioration in their vision. At a 12-month median follow-up period, 11 (19%) patients were dead, 10 (10%) eyes had poor vision (BCVA less than 0.1), and 21 (22%) eyes had BCVA 0.5 or better.

## 4. Discussion

Optic disc swelling can manifest unilaterally or bilaterally, and it arises from a range of diseases. These diseases encompass central nervous system tumors, which not only pose a risk to life but also to vision. Other contributing factors include IIH, ischemic optic nerve disorders, inflammatory processes, exposure to toxic substances, and hereditary optic nerve diseases [12].

The prevalence of these diseases can vary depending on the age of the affected patient. In pediatrics (children and adolescents), the common causes of optic disc swelling include IIH, a condition characterized by elevated intracranial pressure of unclear etiology, brain tumors, such as medulloblastoma, and meningitis [13,14,15]. In young adults (18–40 years), the common causes include idiopathic intracranial hypertension (IIH), particularly in females, and medications such as tetracycline antibiotics, specific oral contraceptives, and vitamin A derivatives [16,17,18]. In middle-aged adults (40–60 years), hypertension and venous sinus thrombosis should be considered [19,20], while in older adults (60+ years), giant cell arteritis and arteriovenous shunts should be considered as major causes for optic disc edema [21,22]. In this study, all the patients had cancer, 45% were less than 20 years old, and 29% were 20–40 years old. In 81% of cases, the optic disc swelling was tumor-related; 52% were due to high ICP secondary to brain tumor (primary or metastatic), 17% had tumor infiltration of the optic nerve, and 12% had compressive optic neuropathy.

In this study, our focus was on investigating the underlying causes of optic disc swelling, specifically in cancer patients. Our findings revealed that in 66% of the cases, optic disc swelling affected both eyes, and high ICP was predominantly attributed to brain tumors, accounting for 70% of the cases. Medication-related side effects were identified as the cause in 20% of cases, with half of these cases being associated with ATRA (Tretinoin, also known as All-Trans Retinoic Acid), a medication used in the treatment of acne and acute promyelocytic leukemia. Notably, the second most common cause of disc edema in our study was tumors infiltrating the optic nerve (17%), while IIH was the underlying cause in only 5% of cases.

IIH is an uncommon disorder of increased ICP characterized by the absence of radiological and laboratory evidence of intracranial pathology [23]. It is sometimes confused with papilledema, which is a more general term for optic disc swelling secondary to any cause of increased ICP, including tumors and other pathologies [24]. IIH is the most common cause of papilledema in the general population, affecting mainly obese women aged 20–44 years, [25] and it is thought to be linked to reduced cerebrospinal fluid absorption [26]. Patients usually experience symptoms like headaches, blurred vision, sensitivity to light, ringing in the ears, or double vision, and are sometimes asymptomatic [27,28]. Diagnosis involves measuring cerebrospinal fluid pressure, using imaging techniques, and checking cerebrospinal fluid chemistry, in addition to finding optic disc swelling [29]. However, only 10% of our patients with high ICP had IIH, and this is because our study focused on cancer patients. In our study, gender was not a risk factor for papilledema because it was related to brain tumors, not IIH. Additionally, 43% of these patients were under 20 years old because many had pediatric brain tumors, which is younger than the typical age range for IIH (20–44 years) [25].

Treatment for high intracranial pressure depends on addressing the underlying cause. In the case of IIH, treatment involves reducing cerebrospinal fluid production using medications like acetazolamide, topiramate, furosemide, or corticosteroids. Weight loss has also been suggested to help improve outcomes [30]. In our study, we used acetazolamide and steroids for all patients with papilledema (but not pseudopapilledema) to prevent optic nerve damage until the underlying tumor was treated or a VP shunt was inserted. This had a positive impact on the visual outcome, as 42% showed an improvement in their visual acuity after treatment.

MRI findings indicative of intracranial hypertension encompass a range of observable manifestations, including an empty sella turcica, optic nerve head protrusion, posterior scleral flattening, augmented perioptic cerebrospinal fluid (CSF), tortuosity of the optic nerve, enlarged Meckel caves, cephaloceles, cerebellar tonsillar descent, and bilateral transverse venous sinus stenosis. At least one of these signs is discernible in approximately half of the individuals with elevated intracranial pressure (ICP) detected clinically [31]. Nevertheless, it is worth noting that MRI scans may appear normal even in the presence of increased ICP with associated papilledema, necessitating a CSF measurement to definitively confirm the diagnosis. In the context of cancer patients, it is imperative to employ pathological CSF analysis to rule out the potential for intracranial tumor metastasis or CSF involvement. In our study, a lumbar puncture was carried out for all patients with suspected high ICP. The pathology of the CSF confirmed the diagnosis in three patients with leukemia infiltration of the optic nerve, in four patients with CNS lymphoma infiltrating the optic nerve, and in three patients with metastasis to the optic nerve.

Optic neuritis is typically the second most common cause of optic disc swelling after high intracranial pressure. However, in our study, optic nerve infiltration by a tumor was the second most common cause, affecting 17% of patients. This infiltration included direct metastasis, leukemic infiltrates, or lymphoma infiltrates. We only identified five patients with inflammatory optic disc swelling in our study, including one with optic neuritis, one with CMV retinitis, and three with radiation-induced optic neuropathy. Typical optic neuritis is often associated with multiple sclerosis (MS), an inflammatory demyelinating disease. Atypical optic neuritis can be due to infections, inflammation, or autoimmune causes [29,30]. The incidence of optic neuritis is around 1–2 per 100,000 people and is more common in young white women [29]. However, it was less common in our study because we focused on cancer patients, who have different characteristics than the general population. Unilateral vision loss is a common symptom of optic neuritis, often accompanied by a relative pupillary defect and color vision changes. Diagnosis typically involves a brain MRI and a cerebrospinal fluid analysis to rule out MS in isolated optic neuritis cases [32,33]. However, in cancer patients, other possible underlying pathologies other than MS should be evaluated such as compressive optic neuropathy, metastatic disease to the optic nerve, and optic nerve infiltration by leukemia or lymphoma (Figure 1).

The Optic Neuritis Treatment Trial found that corticosteroids can improve short-term visual outcomes, but they have limited long-term effects [32,34]. In atypical optic neuritis, treatment depends on the underlying cause, and steroids, along with other immunosuppressive drugs, can improve outcomes, especially in cases of neuromyelitis optica [29,34]. In our study, steroids had a positive effect on various types of optic disc swelling, including papilledema, inflammatory optic neuritis, and infiltrative optic swelling. Steroids work by reducing swelling around the optic nerve, and alleviating pressure until the underlying condition is treated. All patients with pathological optic disc swelling in our study (except the one with CMV retinitis) received systemic steroids, resulting in improved vision in 42% of patients and only 4% experiencing worsening vision. In our study, one woman who had breast ca presented with optic neuritis and was found to have multiple sclerosis. She was treated using a systemic steroids protocol, and her vision was salvaged.

We observed various causes of unilateral optic disc swelling in our patients, including central retinal vein occlusion, central artery occlusion, anterior ischemic optic neuropathy, and radiation optic neuropathy. These cases require tailored diagnosis and treatment to prevent recurrent swelling and disease progression. Non-arteritic ischemic optic neuropathy can also cause optic disc swelling, presenting as painless vision loss in individuals over 50 years old [35]. While it was rare in our study, its reported incidence is 2–10 cases per 100,000 people, and it does not exhibit a gender bias [36]. Risk factors include diabetes, hypertension, smoking, acute hemorrhage, anemia, and low blood pressure [37]. Currently, there is no specific treatment, but various approaches like anticoagulants, vasodilators, and thrombolytics have been attempted with limited success [5].

Paraneoplastic optic neuropathy is a rare condition with an unknown cause. It is believed that antibodies produced in response to tumor-related proteins may harm neurons and glial cells, often involving CRMP-5 IgG antibodies. These antibodies can lead to permanent damage of the nerve function. Paraneoplastic optic neuropathy can manifest not only as optic nerve inflammation but also as retinitis and vitreous cellular reactions [38,39]. In our study, one patient with renal cell carcinoma likely had paraneoplastic optic neuropathy, as they had mild vitritis that improved with steroids. Ideally, suspected cases of PON should be tested for anti-CRMP-5 antibodies, but logistical challenges prevented us from doing so in our setup. Among our patients, three had lung carcinoma: one had brain metastasis with increased intracranial pressure, the second had pseudopapilledema (optic disc drusens), and the third had CMV retinitis. None of them were diagnosed with paraneoplastic optic neuropathy.

Lastly, it is important to differentiate true optic disc swelling from pseudopapilledema, where the optic disc appears swollen without any disease. Conditions like optic disc drusen, myelinated nerve fibers, and high hyperopia can create a pseudopapilledema appearance. Special diagnostic techniques such as autofluorescence, ultrasonography, computed tomography, and optical coherence tomography can help distinguish these conditions [40,41]. Optic disc drusen, composed of calcified hyaline bodies, typically do not require treatment. In our study, four patients had pseudopapilledema, one due to optic disc drusen and three due to high hyperopia. These cases need to be diagnosed to avoid unneeded therapy.

In conclusion, other than eye cancers [42,43,44], systemic different underlying pathologies are recognized as potential sources of optic disc swelling in cancer patients, a condition of significant concern due to its potential to cause vision loss. Early detection plays a pivotal role, and it is essential to conduct a thorough assessment of optic nerve edema in patients experiencing vision loss and symptoms of increased intracranial pressure. It is imperative to promptly administer symptomatic treatment, followed by addressing the underlying cause, to prevent optic nerve damage and vision loss among cancer patients.

## Figures and Tables

**Table 1:** Demographics for 58 cancer patients and visual acuity for 96 eyes with optic disc edema.

		**Number (%)**
Gender	Male	25 (43%)
	Female	33 (57%)
Age	0–20	26 (45%)
	21–40	17 (29%)
	41–60	11 (19%)
	More than 60	4 (7%)
	Mean, Median	38, 36 Years
Laterality	Bilateral	38 (65%)
	Right only	8 (14%)
	Left only	12 (21%)
Visual acuity (decimal value)	At presentation	At last, follow-up
0.8–1.0	39 (41%)	52 (%)
0.5–0.7	19 (%)	21 (%)
0.1–0.5	17 (%)	13 (%)
CF *	9 (%)	6 (%)
HM or PL **	5 (%)	2 (%)
NPL ***	3 (%)	2 (%)
Cannot be assessed	4 (%)	-
Color vision	At presentation	At the last follow-up
Normal	43 (%)	61 (%)
Partially damaged	22 (%)	21 (%)
Total loss	13 (%)	14 (%)
Cannot be assessed	18 (%)	-
Vision acuity change	Improved	40 (42%)
	Stable	52 (54%)
	Deteriorated	4 (4%)

* CF: counting fingers; ** HM or PL: hand motion or perception of light; *** NPL: no perception of light.

**Table 2:** Causes and management for 58 cancer patients with optic disc edema.

		Number of Patients (%)	Number of Eyes (%)	Vision < 0.5 at Diagnosis ^+^
Total		58 Patients	96 Eyes	34 Eyes (35%)
Direct cause	High ICP *	30 (52%)	60 ^&^	8 (14%)
	Infiltration	10 (17%)	14	14 (100%)
	Compression	7 (12%)	8	5 (63%)
	Inflammation **	5 (9%)	5	5 (100%)
	Ischemic ***	2 (3%)	2	2 (100%)
	Psudopapillidema ****	4 (7%)	7	0 (0%)
High ICP *	Total 30 patients			
	Tumor ^&^	21 (70%)	42 ^&^	8 (21%)
	Drugs ^$^	6 (20%)	12	0 (0%)
	Idiopathic	3 (10%)	6	0 (0%)
Infiltration	Total 10 patients			
	Leukemia	3 (30%)	5	5 (100%)
	Lymphoma	4 (40%)	7	7 (100%)
	Metastasis ^#^	3 (30%)	4	4 (100%)
Management	Steroids		53 (91%)	
	Diamox		30 (52%)	
	Chemotherapy ^@^		20 (34%)	
	Radiation therapy		13 (22%)	
	Frequent LP		12 (21%)	
	Surgery (excision/Shunt)		28 (48%)	
	No treatment		4 (7%)	

* ICP: intracranial pressure. ** Optic neuritis 1, CMV retinitis 1, radiation retinopathy 3. *** 1 CRVO, 1 AION. **** One had optic disc drusen, and three had hypermetropia. ^&^ This was the primary CNS tumor in 16 patients and brain metastasis in 5 patients. ^$^ Three patients had high ICP secondary to ATRA (All-Trans Retinoic Acid) and three secondary to systemic steroids. ^#^ The 3 patients with optic nerve metastasis had breast Ca, lung Ca, and retinoblastoma. ^@^ All these 20 patients received systemic chemotherapy and 2 of them received additional intrathecal chemotherapy. **^+^** The visual acuity at diagnosis was assessed for all except 2 very young children with bilateral optic disc swelling due to high intracranial pressure.

## Data Availability

Data are available on reasonable request on demand to the corresponding authors.
